# Dementia Careblazers YouTube: Public Education Tool for Sharing Evidence-Based Dementia Care Information

**DOI:** 10.1093/geroni/igab046.1944

**Published:** 2021-12-17

**Authors:** Natali Edmonds

**Affiliations:** Dementia Careblazers, Phoenix, Arizona, United States

## Abstract

Dementia Careblazers, created and hosted by Dr. Natali Edmonds, board certified Geropsychologist, offers weekly YouTube videos to family caregivers of people living with dementia. These free brief videos provide actionable, evidence-based information and resources focused on dementia caregiving. This virtual modality is particularly relevant for caregivers of people living with dementia given the difficulty family caregivers have in finding supervision and care for the person with dementia in their absence and considering recent health risks through face to face interactions. Furthermore, the free archive of Dementia Careblazers videos allows for access to evidence-based dementia care information at any time, regardless of geographic location or time zone. During this session, Dr. Edmonds will discuss the role YouTube videos play in public education and share tips for starting an evidence-based YouTube channel.

